# Neoadjuvant nivolumab and cabozantinib in advanced renal cell carcinoma in a horseshoe kidney – how to achieve a safe and radical resection? a case report and review of the literature

**DOI:** 10.3389/fonc.2023.1115901

**Published:** 2023-07-14

**Authors:** Anezka Zemankova, Hana Studentova, Andrea Kopova, Tomas Tichy, Vladimir Student, Bohuslav Melichar

**Affiliations:** ^1^ Department of Oncology, Faculty of Medicine and Dentistry, University Hospital Olomouc, Palacky University, Olomouc, Czechia; ^2^ Department of Clinical and Molecular Pathology, Faculty of Medicine and Dentistry, University Hospital Olomouc, Palacky University, Olomouc, Czechia; ^3^ Department of Urology, Faculty of Medicine and Dentistry, University Hospital Olomouc, Palacky University, Olomouc, Czechia

**Keywords:** renal carcinoma, neoadjuvant, immunotherapy, cabozantinib, nivolumab, complete response, horseshoe kidney, case report

## Abstract

**Introduction:**

Neoadjuvant nivolumab and cabozantinib in locally advanced renal cell carcinoma in a horseshoe kidney is a novel therapeutic approach in the preoperative setting.

**Methods:**

We report a case of a 52-year old male who presented with a large inoperable tumor of the horseshoe kidney and achieved major partial radiologic response after neoadjuvant therapy with nivolumab and cabozantinib leading to radical resection of the tumor. The patient remains tumor free on the subsequent follow-up and his renal function is only mildly decreased. The systemic treatment was complicated by hepatotoxicity leading to early nivolumab withdrawal.

**Results:**

Currently, the combination therapy based on immune checkpoint inhibitors and tyrosine kinase inhibitors represents the treatment of choice in treatment-naïve patients with metastatic renal cell carcinoma in any prognostic group. The neoadjuvant treatment approach is being tested in prospective clinical trials and results are eagerly awaited. Renal cell carcinoma in a horseshoe kidney is an uncommon finding that is always challenging. Additionally, management guidance in this patient population is lacking. In some patients neoadjuvant therapy could be the only way to preserve kidney function. The initial treatment strategy should be individualized to patient needs aiming at the radical resection of the primary tumor as the only chance of getting the tumor under control in the long term.

**Conclusion:**

Herein, we highlight the feasibility of neoadjuvant systemic therapy with nivolumab and cabozantinib allowing the subsequent performance of radical tumor resection with negative margins in a patient with advanced renal cell carcinoma in a horseshoe kidney, removing the primary tumor while sparing the patient from lifelong dialysis.

## Introduction

In recent years, the use of immune checkpoint inhibitors (ICIs) has revolutionized the therapeutic landscape of metastatic renal cell carcinoma (mRCC) (1–5). Undoubtedly, immunotherapy denotes a great opportunity to control the disease in patients from a long-term perspective. The widespread use of ICIs across various tumor types represents an unprecedented advancement in the therapy leading to cancer cell elimination. However, less advance has been made in terms of identification of patients who would derive benefit from immune targeted approach.

The current first line strategy in mRCC comprises mainly a combination of an ICI plus tyrosine-kinase inhibitor (TKI) based on prior demonstration of the benefit of these combinations. One such example is the combination of an anti-PD1 antibody nivolumab with TKI cabozantinib which has shown superior efficacy compared with sunitinib monotherapy in the first-line setting in patients with mRCC regardless of their prognosis according to the International Metastatic Database Consortium (IMDC) Risk Score for RCC, introducing a shift in the common therapeutical practice of the first-line treatment (3). Moreover, combination immunotherapy with a PD-1 inhibitor and TKIs have shown high complete and overall response rates in the metastatic setting. Nevertheless, the efficacy of the combination in the preoperative setting needs to be evaluated (6).

Localized renal cell carcinoma (RCC) can be inoperable for several reasons including solitary kidney or locally advanced disease. Data on optimal therapeutic strategy in this patient population are scarce. The potential of the neoadjuvant treatment approach in this patient population lies in downsizing the primary tumor, enabling radical tumor resection (7).

We describe a case of a patient with locally advanced inoperable RCC of a horseshoe kidney treated with neoadjuvant nivolumab and cabozantinib leading to radical resection of the primary tumor 7 months later.

## Case description

**Figure 1:**
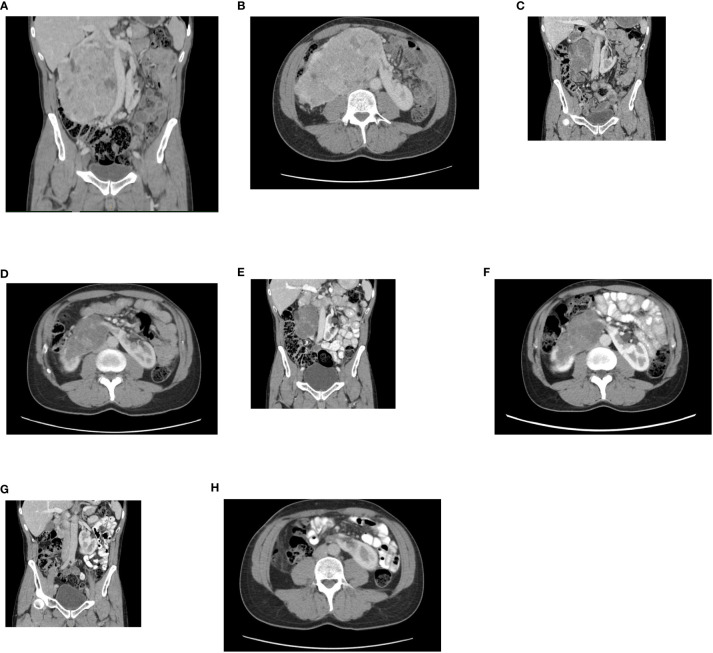
**(A)** Initial contrast-enhanced CT. A horseshoe kidney with a bulky tumor affecting a large part of the right kidney, a coronal plane. **(B)** An axial plane. **(C)** Follow-up contrast enhanced CT. Partial regression of the tumor after, 2 month from therapy initiation, a coronal plane. **(D)** An axial plane. **(E)** Follow-up contrast enhanced CT. Partial regression of the tumor, 5 months from therapy initiation, a coronal plane. **(F)** An axial plane. **(G)** Follow-up contrast enhanced CT. Complete radiographic regression of the tumor, a coronal plane. **(H)** An axial plane.

A 52-year-old Caucasian male, non-smoker, with an Eastern Cooperative Oncology Group (ECOG) status 0 with no serious comorbidities presented in October 2021 with anemia and hematuria. The abdominal CT scan revealed a horseshoe kidney, a bulky tumor at the junction of the lower kidney poles affecting a large part of the right kidney. The dimensions of the tumor were 150x120x100 mm (**Figures 1A, B**). The tumor was located close to large abdominal vessels. The chest CT and bone scan showed no evidence of metastatic disease. Upon review of imaging during the multidisciplinary genitourinary board, it was concluded that the tumor was unresectable due to its size and proximity to the renal vasculature. Moreover, the risk of irreversible kidney injury resulting in the need of lifelong dialysis was extremely high. Ultrasound-navigated biopsy of the tumor was performed. Histology subsequently revealed grade 1 clear cell renal cell carcinoma (ccRCC). After an in-depth discussion with the patient, nephrectomy was not indicated and the patient opted to proceed with off-label systemic treatment consisting of nivolumab and cabozantinib.

**Figure 2:**
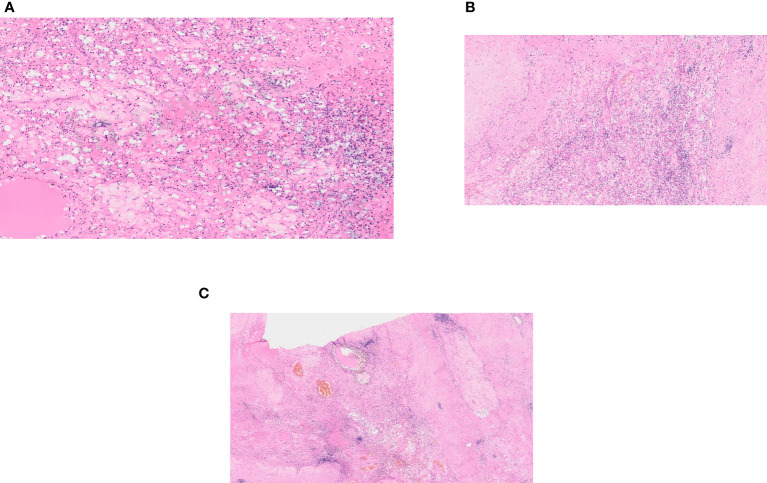
Histological examination. **(A)** A regressively changed clear cell renal cell carcinoma. **(B)** A regressively changed clear cell renal cell carcinoma in detail. **(C)** A regressively changed clear cell renal cell carcinoma, hyalinosis and stromal edema.

The combination therapy with nivolumab (240 mg intravenously every two weeks) and cabozantinib 40 mg orally daily was started in December 2021. In January 2022, 6 weeks after therapy initiation, the patient developed grade 3 hepatotoxicity according to Common Terminology Criteria for Adverse Events (CTCAE) version 5.0 manifested by elevation of aspartate aminotransferase (AST) and alanine aminotransferase (ALT) with bilirubin levels within normal limits. Other potential causes of hepatotoxicity including infections or obstruction were ruled out. The therapy was stopped immediately and corticosteroid administration with prednisolone 1mg/kg orally was initiated with close monitoring of liver enzymes and other related parameters. Within a week of therapy, slow lowering of corticosteroid dose was possible because of a decrease in AST and ALT levels to grade 1 suggesting immune-related toxicity. In February 2022, the restaging CT scan was performed showing tumor size reduction to 90x60x100 mm, i.e. partial regression by Response Evaluation Criteria in Solid Tumors (RECIST) 1.1 was achieved (**Figures 1C, D**). Since the surgical intervention was not feasible yet, it was decided to continue with systemic treatment for additional 3 months. However, nivolumab had to be permanently discontinued due to the history of grade 3 hepatotoxicity, and single agent cabozantinib was administered since March 2022. The standard cabozantinib dose of 60 mg daily had to be reduced to 40 mg daily because of hand-foot syndrome. In May 2022, the CT scan showed an additional decrease in the tumor size (90x55x95mm) with no evidence of distant metastases. The most marked effect of the therapy was noticed at the start of the treatment, with only mild reduction of the tumor in the last 3 months (**Figures 1E, F**). Taking this into consideration, the multidisciplinary tumor board team decided to proceed with radical tumor resection. In June 2022, after one-month washout of cabozantinib, the radical resection, including a right heminephrectomy of the horseshoe kidney was performed successfully without any postoperative complications. A significant decrease in estimated glomerular filtration (GF) (>25%) was noted, but the value of GF remained above 50 ml/min (chronic kidney disease stage 3a). Final histological analysis showed regressively changed (ccRCC) (**Figures 2A–C**), pathologic TNM classification was pT2a with negative margins evaluated as a major partial response.

**Figure 3:**
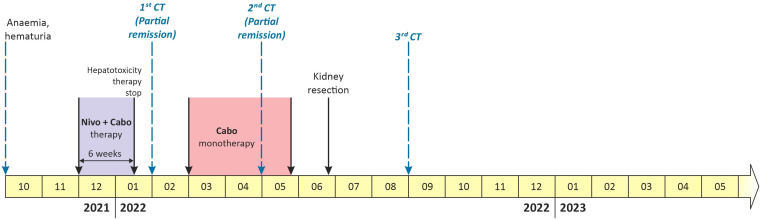
A timeline showing relevant data from the patient case report.

In September 2022, a follow up CT scan confirmed complete radiographic remission, and no further systemic therapy was offered after the operation (**Figures 1G, H**). The patient remains asymptomatic and disease-free 20 months after the initial diagnosis. All relevant data are depicted in a timeline (**Figure 3**).

## Discussion

We report here an excellent outcome of the combination of nivolumab plus cabozantinib given in the neoadjuvant setting in a patient with locally advanced inoperable RCC in a horseshoe kidney. The therapy led to significant tumor shrinkage and subsequent successful surgical resection of the tumor with durable remission. To the best of our knowledge, this is the first report of neoadjuvant nivolumab plus cabozantinib, enabling radical tumor resection due to therapy-induced downsizing of the primary tumor, reported in the literature.

Neoadjuvant therapy remains under investigation and no systemic therapy in localized or locally advanced RCC has been FDA-approved so far. The successful use of a preoperative treatment approach to facilitate surgical resection using multiple tyrosine kinase inhibitors (MTKIs) was reported only in anecdotal case reports (8, 9). In several phase 2 trials addressing the neoadjuvant strategy low complete response rate has been typically observed (10–12). Neoadjuvant cabozantinib in patients with unresectable locally advanced RCC facilitating nephrectomy has also been reported (13–15). Combination therapy of nivolumab and cabozantinib yielded the greatest reduction in the primary tumor size in a retrospective study (16).

Antitumor response promoted by ICIs via PD-1/PD-L1 inhibition potentially induces a long-term effect by eliminating of metastatic clones. Concerning immunotherapy in the preoperative setting, in 2021 Gorin et al. published data on high-risk nonmetastatic RCC patients given preoperative nivolumab (only 3 doses of nivolumab, 240 mg, administered intravenously) with only 1 patient out of 17 having pathological response with immune-related features in the removed kidney (17). The PROSPER RCC trial presented at ESMO 2022 did not show any improvement in recurrence free survival in patients with a high risk of recurrence in the arm with perioperative nivolumab (10 doses of nivolumab, 480 mg, 1 dose administered prior to surgery followed by 9 doses postoperatively) (18). Results from further clinical trials currently underway, including dual-agent immunotherapy, may elucidate the optimal therapeutic strategy in this patient population (19, 20). In Checkmate-9ER, the overall response rate in the whole population was 55.7%, however, efficacy data of locally advanced patients from the trial have not been published yet (3).

The therapy preference should always be carefully considered and discussed in patients with a solitary kidney or significantly impaired renal function where kidney function preservation is at risk. Regarding the large inoperable tumor mass in this case, we decided to initiate the combination strategy of nivolumab with cabozantinib to attempt downsizing the tumor. One of the critical moments of the whole process is finding a strategy that would preserve maximum of functional renal parenchyma, and failure to do this adversely affects the patient´s prognosis

The tumor size reduction was rapid at the beginning of the therapy when the combination of both drugs was given. The tumor diameter decreased by 30% after 2 months of cabozantinib therapy with nivolumab, while an additional 4% decrease was obtained during single-agent cabozantinib therapy. The pathologist described the tumor as 85x87x50 mm in size, but only a small portion of the tumor consisted of viable tumor cells. The discrepancy between the radiological and actual tumor size has been previously described as viable tumor cells might represent only a small proportion of the remaining mass (21).

Management guidance on tumors arising from a horseshoe kidney remains anecdotal owing to the rarity of this presentation. Only case reports or small case series provide clinical data on therapeutic management specific to this particular presentation. The variable anatomy of a horseshoe kidney, aberrant vasculature with accessory arteries and branches arising from arteries other than the aorta, and the complexity of the tumor renders surgery highly demanding (22). In order to optimize the final treatment outcome, preoperative planning should always precede.

Various histopathological subtypes of renal abnormalities have been described in association with renal tumors in a horseshoe kidney, hence a tumor biopsy preceding the operation is warranted.

In the current case, no complications associated with the surgery or wound healing disturbances were noted. Wound healing problems resulting from TKI therapy have been previously published (23), as were fibrotic changes induced by ICIs and discovered during the surgery (24). A combination of both approaches may become a challenge for the operating surgeon.

Not only efficacy but also safety is of concern when we manage a patient with localized disease considering a future surgical procedure. Identifying the optimal therapy at the very beginning is crucial as novel potent therapies emerge. The patient in the present case experienced grade 3 hepatotoxicity leading to ICI termination followed by single agent TKI. In the Checkmate-9ER trial 5.6% patients discontinued both agents and nearly 20% patients permanently stopped the combination therapy and continued with single agent therapy due to adverse events (3). Toxicity may develop later during the course of therapy such as the case of a RCC patient who developed hypophysitis 9 months after ICI therapy initiation that manifested during the postoperative course following cytoreductive nephrectomy (25). Adverse events induced by ICIs may be unpredictable and patients whenever received immunotherapy should be carefully followed for potential side effects.

## Conclusion

The present report demonstrates the potential of neoadjuvant nivolumab and cabozantinib therapy to downsize an initially inoperable RCC leading to subsequent radical surgical resection. The neoadjuvant approach with nivolumab and cabozantinib may be a viable option for patients with initially inoperable RCC, including patients with a solitary kidney.

## Data availability statement

The original contributions presented in the case report are included in the article/supplementary material. Further inquiries can be directed to the corresponding author.

## Ethics statement

Ethical review and approval was not required for the case report in accordance with the local legislation and institutional requirements. Written informed consent was obtained from the individual for the publication of any potentiall identifiable images or data included in the article.

## Author contributions

AZ: writing and revision of the manuscript, collected data. HS: conceptualization, supervision, writing and revision of the manuscript. AK: collected data, revision of the manuscript. TT: revision of the manuscript. VS: revision of the manuscript. BM: revision of the manuscript. All authors contributed to the article and approved the submitted version.
